# Advancing Gender Equality in Australian Healthcare Leadership: Why National Targets Matter and What It Means for Healthcare Employers

**DOI:** 10.5694/mja2.70300

**Published:** 2026-09-16

**Authors:** Belinda Garth, Nicole Pope, Jenny Proimos, Helena J. Teede

**Affiliations:** ^1^ Monash Centre for Health Research and Implementation Monash University Melbourne Victoria Australia; ^2^ Monash Health Melbourne Victoria Australia

**Keywords:** health services, medical colleges, medical legislation, women physicians

## Abstract

Women comprise the majority of Australia's healthcare workforce yet remain underrepresented in leadership. This structural inequity has direct consequences for workforce sustainability and patient outcomes. Australia's new gender equality target‐setting requirements, which create binding obligations for large employers, including major healthcare services, offer a timely mechanism to address this structural inequity. In this article, we explain why gender equality targets matter for healthcare, outline who must comply and what is required and describe how health services and training settings can embed targets within governance, clinical accountability and workforce systems. We introduce the Monash Learning Health System Maturity Matrix as a practical tool for aligning targets with organisational maturity and argue that, when implemented well, gender equality targets go beyond compliance, building the institutional resilience and culture that retains talent and delivers better care.

## 1 Introduction

Australia's healthcare system faces a structural challenge with direct consequences for both patients and workforce. Women comprise the majority of the healthcare workforce yet remain underrepresented in leadership, where decisions about care delivery and workforce priorities are made [1, 2, 3]. The intersection of gender with race and ethnicity further compounds this inequality [4, 5].

This lack of representation has tangible system‐level effects [6, 7]. When leadership does not reflect the workforce, frontline experience risks being overlooked, staff engagement diminishes and capacity to identify and respond to emerging challenges is reduced [8]. Organisational cultures that constrain career progression contribute to attrition, undermine sustainability and limit diverse perspectives in clinical and organisational decision‐making [9].

These consequences are not theoretical. Evidence links leadership diversity to reduced patient mortality and improved organisational performance, workforce well‐being, quality, safety and equity, particularly for women and children [6, 8, 10]. Yet, at the current pace of change, gender equality remains nearly a century away [11], an untenable trajectory for a sector where inequity directly affects workforce sustainability and patient outcomes [8].

Against this backdrop, setting gender equality targets is widely recognised as an effective mechanism to drive measurable progress [12, 13]. Targets are specific, measurable objectives with defined timeframes, generally set at organisational discretion [14] and typically aspirational, carrying minimal consequences if unmet. Quotas, by contrast, impose binding obligations set by an external authority, backed by formal penalties for non‐compliance [12].

Australia's policy landscape has shifted significantly, creating a critical opportunity to accelerate progress. Commitments to the United Nations Sustainable Development Goal Five (SDG 5) [15] and the Working for Women Strategy [16] have informed new national approaches to gender equality. In this context, the National Health and Medical Research Council (NHMRC) and partner‐funded Advancing Women in Healthcare Leadership (AWHL) initiative was established to coproduce and scale system‐ and organisational‐level interventions to improve women's leadership progression [17]. AWHL sits within the Monash University‐led Partnership Centre for Gender Equality and Leadership Advancement and includes over 28 partners across government, health services, professional associations, research institutes, academia and the not‐for‐profit sector.

Consistent with our submission to the Senate Inquiry into the Workplace Gender Equality Amendment (Setting Gender Equality Targets) Bill 2024, advancing gender equality in leadership and beyond requires shifting responsibility from individual to system‐ and organisational‐level action. Regulatory approaches are most effective when anchored in explicit goals, such as targets, combined with accountability rather than reporting alone [9]. Evidence is clear that organisational change is strengthened when leadership commitment and accountability are coupled with five core organisational strategies spanning policy, culture, measurement, mentoring and embedded leadership development [9]. Australia's new gender equality target‐setting requirements [18] therefore provide a timely opportunity for healthcare services to embed these evidence‐based approaches and accelerate progress towards more equitable leadership.

In this article, we aim to (i) introduce the new national gender equality target‐setting requirements and their relevance to healthcare services and training settings; (ii) show how targets drive change in practice; and (iii) offer practical guidance on implementation.

## 2 Regulatory Context: Which Employers Must Set Targets?

### 2.1 Australian Private‐Sector and Commonwealth Public‐Sector Employers

From April 2026, gender equality targets became a regulatory requirement for Australian private‐sector and Commonwealth public‐sector employers who directly employ 500 or more employees and report to the Workplace Gender Equality Agency (WGEA) [18]. Under these requirements, employers must:

- set three gender equality targets from WGEA's legislated menu of 19, which include numeric and action targets; and
- commit to achieving these targets over a 3‐year cycle.

At the end of 3 years, employers must meet or demonstrate improvement against each target. This represents a form of mandated target‐setting: the obligation to set and pursue targets is binding, but employers retain flexibility in selecting which targets to pursue. Unlike quotas, there are no hard financial or legal penalties for failing to meet the targets themselves. Instead, the consequences for non‐compliance are reputational, principally through public naming by WGEA.

Employers with 100 or more employees and fewer than 500 will continue to report to WGEA but are not required to set targets, whereas those with fewer than 100 employees are exempt from WGEA reporting.

### 2.2 Victorian Public‐Sector Employers

**FIGURE 1:**
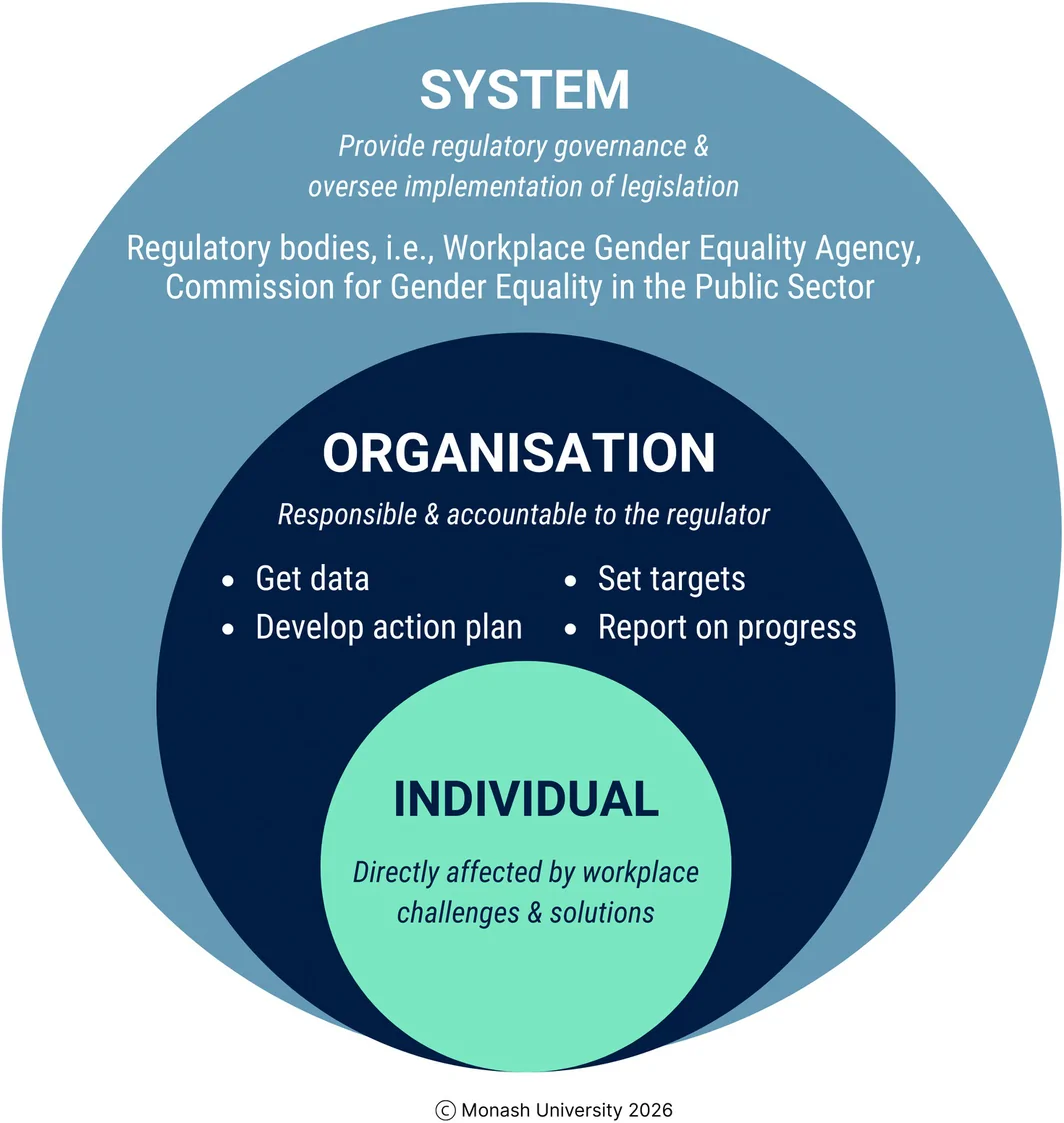
Conceptual framework of stakeholder roles in workplace gender equality target‐setting.

Gender equality targets are not mandated for Victorian public‐sector organisations, which report instead to the Commission for Gender Equality in the Public Sector (CGEPS) under the *Victorian Gender Equality Act 2020* [19]. Although targets are not required, the Commission has released early findings from research examining how gender equality targets could be applied within the Victorian public sector. Aligned with our evidence synthesis on effective organisational change strategies [9], the CGEPS's findings identify enabling conditions for success, which include robust data systems, intersectional approaches, strong leadership commitment and active staff engagement and communication [20]. Figure 1 summarises the key stakeholders and their roles in gender equality target‐setting.

## 3 Voluntarily Setting Gender Equality Targets: Why It Matters

Even where gender equality targets are not mandated, adopting them remains best practice. Voluntary targets enable employers to accelerate progress, signal accountability and foster the cultural and structural conditions needed for sustained change, including identifying priority gaps, improving workplace culture and enhancing transparency in governance [9, 14]. Evidence shows that organisations with clearly defined targets (supported by accountability mechanisms) achieve more consistent and sustained outcomes than those relying on unstructured approaches [12, 21].

The tertiary education sector offers a compelling example. Through Science in Australia Gender Equity (SAGE), which is an AWHL partner, participating universities and research institutes report smaller gender pay gaps and greater representation of women in senior roles compared with non‐subscribers [21], demonstrating that structured, accountable approaches outperform goodwill alone.

## 4 How Targets Enable Change

To accelerate and sustain progress, targets should not be treated as a stand‐alone administrative burden. Evidence shows they should be aligned with and embedded within governance, clinical accountability and workforce systems [22]. In hospital and health service settings, targets can be aligned with transparent and publicly available board performance metrics, incorporated into clinical governance frameworks and assigned clear executive ownership (e.g., Chief Medical Officer, Chief Nursing Officer or People and Culture leads). In primary care and private sector organisations, they can be built into accreditation processes, workforce strategies and partnership agreements. Education and training providers can embed targets within leadership, promotion pathways and trainee selection.

## 5 Practical Implementation: Matching Targets to Organisational Maturity

Selecting the right targets matters as much as setting them. Health services vary considerably in their data infrastructure, leadership commitment and implementation capacity. Targets that are poorly matched to organisational maturity risk being fragmented, underresourced or unsustainable. Understanding where a health service sits on this spectrum is therefore an essential first step.

The Monash Learning Health System Maturity Matrix (LHS‐MM) [23] provides a structured, evidence‐based approach to this assessment and is applicable to all health system challenges, including gender inequality. Grounded in the Learning Health System (LHS) model, which is a core pillar of the NHMRC Research Strategy 2026–2036 [24], it integrates research, data and practice to support continuous improvement. Organisations self‐assess across four evidence domains:

- *stakeholder evidence*: lived experience and priorities of individuals with a stake in the problem and solutions;
- *research evidence*: high‐quality literature and best practice;
- *data and current practice evidence*: existing organisational data and sector activity; and
- *implementation evidence*: translating evidence from domains 1–3 into practice and evaluating impact.

**FIGURE 2:**
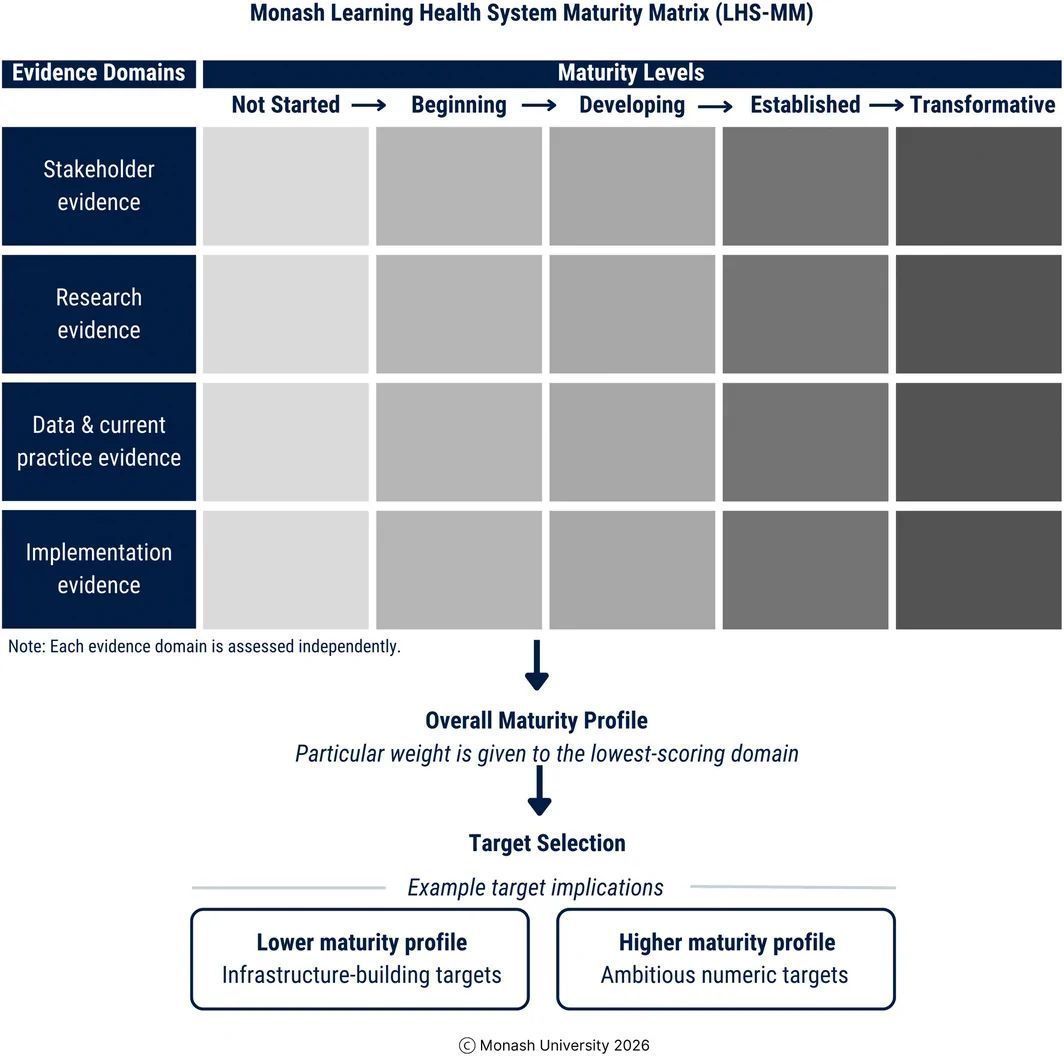
Application of the Monash Learning Health System Maturity Matrix (LHS‐MM) [23] to gender equality target selection. Four evidence domains are assessed across five maturity levels. Target selection is guided by the overall maturity profile, with particular weight given to the lowest‐scoring domain.

Together, these domains provide a systematic and continuously integrated pathway from problem identification to sustained improvement. Maturity is rated across five levels, from ‘not started’ to ‘transformative’, to inform target selection (Figure 2). As a general rule, target selection follows a service's overall maturity profile, with particular weight on its lowest‐scoring domain, since that is the capability most in need of building before more ambitious commitments become realistic. For example, a service with siloed data and early‐stage systems might prioritise infrastructure‐building targets, whereas one with integrated data and implementation capacity can credibly commit to ambitious numeric targets. Throughout, attention to intersectional, disaggregated data helps ensure targets advance equity broadly and avoids situations where aggregate gains mask persistent inequities among groups experiencing compounded disadvantage.

In practice, the LHS‐MM functions as a diagnostic process that complements formal gender equality reporting and can be conducted as a focused workshop with key stakeholders across the executive, People and Culture, workforce data analytics and clinical operations. Repeating the assessment annually across the reporting cycle of a regulator such as WGEA makes progress visible. In this way, gender equality target‐setting becomes not a stand‐alone exercise but part of a continuous, system‐level improvement process embedded within routine organisational practice.

## 6 Conclusion

Diverse healthcare leadership is essential for quality care. Gender equality targets offer a timely opportunity to address structural inequity in Australian healthcare (strengthening governance, increasing leadership diversity and embedding equitable practices) particularly when supported by evidence‐based implementation approaches.

Practical support is available through WGEA's Targets Hub and Data Explorer, CGEPS guidance for Victorian employers and AWHL's LHS‐MM and implementation masterclasses and mentoring programs [14, 19, 25].

When health services and training institutions invest in gender equity, they go beyond compliance, building the institutional resilience and culture that retains talent, reduces harm and delivers better outcomes for patients and communities.

## Author Contributions

**Belinda Garth:** conceptualisation, writing (original draft). **Nicole Pope:** writing (review and editing). **Jenny Proimos:** writing (review and editing). **Helena J. Teede:** conceptualisation, writing (review and editing), funding acquisition.

## Funding

The Advancing Women in Healthcare Leadership (AWHL) national initiative is supported by funding from two National Health and Medical Research Council (NHMRC) Partnership Grants (APP1198561 and 2018718) and partner contributions. The NHMRC had no role in preparation or reporting of this article. Helena J. Teede receives funding from an NHMRC Fellowship.

## Disclosure

Not commissioned; externally peer reviewed.

## Conflicts of Interest

The authors declare no conflicts of interest.

## Data Availability

The authors have nothing to report.
